# Susceptibility-related differences in the quantity of developmental stages of *Myxobolus* spp. (Myxozoa) in fish blood

**DOI:** 10.1371/journal.pone.0204437

**Published:** 2018-09-21

**Authors:** Dóra Sipos, Krisztina Ursu, Ádám Dán, Dávid Herczeg, Edit Eszterbauer

**Affiliations:** 1 Institute for Veterinary Medical Research, Centre for Agricultural Research, Hungarian Academy of Sciences, Budapest, Hungary; 2 Veterinary Diagnostic Directorate, National Food Chain Safety Office (NFCSO), Budapest, Hungary; University of Minnesota, UNITED STATES

## Abstract

Here, we investigated the early development of two closely related myxozoan parasites, the highly pathogenic *Myxobolus cerebralis*, the causative agent of the whirling disease in salmonids, and *Myxobolus pseudodispar*, a common, non-pathogenic parasite of cyprinids. The aim of our study was to examine under *in vivo* laboratory conditions whether fish blood is involved in the intrapiscine development of the two parasite species and investigate if there is dissimilarity between the parasite infection intensity in blood and if it varies in terms of host susceptibility and parasite pathogenicity. Highly susceptible, less susceptible and non-susceptible hosts were involved. Blood samples were taken 1 day, 1 week and 1 month post exposure to *M*. *cerebralis* and *M*. *pseudodispar*, respectively. The prevalence and infection intensity was estimated by parasite-specific quantitative real-time PCR. Although previous findings assumed that *M*. *cerebralis* might escape from host immune system by migrating via peripheral nerves, our experimental results demonstrated that *M*. *cerebralis* is present in blood during the early stage of intrapiscine development. For the non-pathogenic *M*. *pseudodispar*, the highest infection prevalence was found in the original host, common roach *Rutilus rutilus*, whereas the highest infection intensity was detected in rudd *Scardinius erythrophthalmus*, a “dead-end” host of the parasite. The presence of *M*. *pseudodispar* developmental stages in the blood of both susceptible and non-susceptible cyprinids suggests that the susceptibility differences remain hidden during the early stage of infection. Our findings supply further evidence that host specificity is not determined during the early, intrapiscine development involving the vascular system. Furthermore, we found remarkable differences in the infection dynamics of the two parasite species examined, possibly due to their distinct pathogenicity or variations in adaptive capabilities to immune components in host blood.

## Introduction

Myxozoans represent a diverse part of phylum Cnidaria with 64 genera and more than 2200, mostly non-pathogenic species [1]. Their largest genus is *Myxobolus* Bütschli, 1882 with more than 900 species [2,3]. Myxozoans have a two-host life cycle first described for *Myxobolus cerebralis* Hofer, 1903 by Wolf and Markiw [4]. Myxozoans have two-host life cycle. The myxospore of parasite infects the invertebrate host (e.g. annelid), and the actinospore form penetrates the vertebrate host (mostly fish)[5]. Myxozoans are strictly host-specific, most of them are only able to infect a single or closely related species [6]. For histozoic species, the sporogonic phase of development is tissue-specific and related to a particular organ of the vertebrate host [6].

*Myxobolus cerebralis* is a highly pathogenic representative of Myxozoa and the causative agent of the whirling disease that inflicts considerable economic loss in aquaculture [7]. There is no effective treatment against whirling disease, however, a newly developed therapeutic concept based on RNA interference could be promising [8]. The parasite has a relatively broad spectrum of host species among salmonids. Rainbow trout *Oncorhynchus mykiss* has the highest susceptibility to the parasite and whirling disease, whereas the infection in the original host, brown trout *Salmo trutta* m. *fario* exhibits less severe symptoms [9]. Additionally, the susceptibility of the host is influenced by the fish strain and inbreeding levels [10–12].

The intrapiscine development of *M*. *cerebralis* starts with the attachment of actinospores to the gill epithelium or to the fin or skin epidermis of the host [13,14]. Previous, histologic and electronmicroscopic examinations of experimentally exposed fish revealed essential details regarding the intrapiscine development of *M*. *cerebralis* [13]. Results of these examinations showed that after the actinospore successfully attaches to the fish, the sporoplasms of the parasite penetrate and multiply mitotically in the epithelial cells of the host. Approximately 2 days post exposure (p.e.), the secondary cells of sporoplasms migrate to the subcutis, then move to the cells of nerve tissues within the peripheral nervous system, and a few weeks p.e., they reach the cartilaginous tissue of central nervous system, where sporogony takes place. El-Matbouli et al. [13] did not find any evidence that *M*. *cerebralis* occurs in blood during the route of migration within the host.

*Myxobolus pseudodispar* Gorbunova, 1936 is genetically more diverse than the majority of the myxozoans. The intraspecific difference in small subunit ribosomal DNA (SSU rDNA) sequences is up to 5% even if the spore morphology and tissue preference is identical, which raises the possibility that *M*. *pseudodispar* is a species complex [15]. It was originally found to infect the common roach *Rutilus rutilus* [16], however, myxospores have been found in common bream *Abramis brama*, white bream *Blicca bjoerkna*, rudd *Scardinius erythrophthalmus*, bleak *Alburnus alburnus* and the European chub *Squalius cephalus* [15,17,18]. *M*. *pseudodispar* forms intracellular cysts in the skeletal muscle of fish, but it is non-pathogenic. When myxospores mature, plasmodia may rupture, and some myxospores may reach the kidney via the blood stream (carried by macrophages), and be released into the environment [18–20]. Supporting this observation, mature myxospores of *M*. *pseudodispar* were also detected in the capillary network of gills [17–19].

Learning how myxozoans successfully invade vertebrate hosts is crucial to understand the epidemiology of diseases caused by the members of this taxon. It is also fundamental for studying their life cycles, understanding host specificity and elucidating the key influencing factors of myxozoan development [21–22]. Kallert et al. [23] showed that sequential chemical and mechanical stimulation were required to discharge the polar capsules of *M*. *cerebralis* actinospores, the initial step of the invasion of vertebrate host. Furthermore, actinospore behaviour during invasion is rather non-specific, as the parasites reacted to the mucus of both susceptible and non-susceptible host species [24]. These important milestones in myxozoan research clearly underlined that in several species, host specificity is not a result of parasite choice.

Although fish mucus is a potent defensive barrier, Kallert et al. [25] proved with *in vitro* inactivation assays that sporoplasms are able to penetrate susceptible fish host tissue without being affected by host immune factors, that are present in fish mucus (e.g. lysozyme, antimicrobial peptides or complement). Studies show that mucus from both susceptible and non-susceptible fish is ineffective in breaking down actinospore sporoplasms of *M*. *cerebralis*, *Henneguya nuesslini* Schuberg & Schroder, 1905 and *M*. *pseudodispar* [25]. However when exposed to blood sera of the same fish species, sporoplasms of these parasite species showed a significant increase in cellular breakdown in non-susceptible host serum relative to serum from susceptible host fish. For *H*. *nuesslini* and *M*. *pseudodispar*, the effectivity of serum from non-susceptible hosts decreased considerably over time. *Myxobolus cerebralis* was the exception, the primary and secondary sporoplasm cells were affected by the serum of a susceptible host in a much shorter time than the other two species examined. To date, little is known on the fate of early invaded stages in non-susceptible hosts before and after the primary cell of sporoplasm disintegrates. Observations on the early development of *M*. *cerebralis* in susceptible and non-susceptible hosts provided insights on factors that may influence invasion. For instance, challenges of non-salmonid fish and the amphibian *Lithobates pipiens* (formerly *Rana pipiens*) with *M*. *cerebralis* have demonstrated specificity towards salmonids at a very initial developmental stage [26], with only few or no early developmental stages detected in non-susceptible fish tissues.

The aim of our study was to gain further knowledge on the host specificity of histozoic myxozoan species possessing different level of pathogenicity. By measuring infection prevalence and infection intensity using quantitative real-time PCR, we examined experimentally (1) whether fish blood was involved in the early intrapiscine development of the highly pathogenic *M*. *cerebralis* and the non-pathogenic *M*. *pseudodispar* species, and (2) we investigated if there were differences among parasite loads in the blood of fish species in terms of host susceptibility and parasite pathogenicity.

## Methods

### Source and maintenance of parasites and fish

The myxozoan parasites *M*. *cerebralis* and *M*. *pseudodispar* lineage GER (latter one characterized by Forró & Eszterbauer [15]) used for exposure trials originated from the life cycles maintained in the laboratory of the Institute for Veterinary Medical Research, Budapest, Hungary since 2007, as described by Eszterbauer et al. [5]. The parasite spores (both myxospores and actinospores) were regularly controlled by microscopy and DNA sequencing to exclude any possible contamination. Contaminant myxozoans were not detected in any case.

Salmonids, rainbow trout *Onchorhynchus mykiss* (Kamloops strain) and brown trout *Salmo trutta* m. *fario* were obtained from the Lillafüred Trout Hatchery in Hungary. Trout fry were kept in a parasite-free environment in the hatchery, and transported to the laboratory about a week after hatching, in “yolk-sac stage”. Prior to exposure, fish were kept in aerated, water flow-through aquaria at 15°C.

The fertilized eggs of cyprinids, common roach, rudd, gibel carp *Carassius gibelio*, and common bream originated from cyprinid fish farms at Dinnyés, Attala and Hortobágy in Hungary. All fertilized eggs were transported to the laboratory before hatch. Fish were reared under parasite-free laboratory conditions in aerated aquaria at 20±2°C. All fish species were fed with commercial fish pellet (Aller Aqua, Denmark) *ad libitum*.

### Experimental design

Three exposure trials were performed, one with *M*. *cerebralis* and two with *M*. *pseudodispar*. Parasite actinospores (triactinomyxons; TAMs) of both species were harvested by filtering the water from the culture containers through 20 μm nylon mesh. TAMs used for the infection trials were less than 2 days old [5]. Naïve fish were exposed individually to 5000 freshly filtered TAMs/fish in 500 ml de-chlorinated tap water at 15°C for salmonids and 20°C for cyprinids, for 3 hours. Thirty-four fish specimens per species were exposed. Non-exposed control fish were kept under the same conditions with the exception of TAMs. After exposure, fish were transferred into aquaria and separated by species. Samples were taken at three time points in all exposure trials: 1 day, 1 week, and 1 month p.e. For the latter two time points, two extra specimens (i.e. 12 instead of 10) per group were added in order to compensate for possible mortality during the experiment. During sample collection, fish were anaesthetized with 200 mg/l tricaine-methanesulfonate (MS222, Sigma, Germany). Whole blood was taken from the caudal vein of every individual, and all needles contained heparin (TEVA, Hungary) as an anticoagulant. Blood samples were stored at -20°C until further molecular use.

For *M*. *cerebralis* exposure, two months old rainbow trout and brown trout fry were used. The average size of fish was 3.5 cm in length. For *M*. *pseudodispar*, two exposure trials were performed using fish of different age and/or species. In *M*. *p*. experiment #1, common roach, gibel carp and rudd were one-year old. In *M*. *p*. experiment #2, two-year old common roach and rudd, and three-year old common bream specimens were used. The average size of year 1+ cyprinids was 4 cm in length. Cyprinids of year 2+ and 3+ were approximately 6 cm long. Due to technical difficulties that appeared prior to the exposure trial, the number of available common bream specimens was reduced, therefore the sampling at 1 month p.e. was skipped for common bream in experiment #2. Blood smears were prepared from all exposed fish in *M*. *p*. experiment #2, and stained with MGG Quick Stain (modified Giemsa) according to the manufacturer’s recommendations (Bio-Optica, Italy).

National Scientific Ethical Committee on Animal Experimentation provided approval for the animal experiments (No. XIV-I-001/1326-4/2012 and PEI/001/4087-4/2015). Animal welfare was a high priority, especially while handling experimental fish. Fish were kept under laboratory conditions. To maintain proper oxygen levels in glass tanks, water was aerated constantly. Appropriate water quality was achieved using biological filters and continuous water flow-through. Fish densities were determined depending on fish size and age. Fish were fed with commercially available fish pellet that corresponded to each species. The frequency of feeding was 1–2 times a day "*ad libitum*", depending on the age of fish. Glass tanks were lightly illuminated for 12 hours a day, and partial shielding and hiding tubes were provided to fish. The well-being of fish was monitored twice a day. *M*. *pseudodispar* infection caused no clinical signs or illness to any fish species examined. For *M*. *cerebralis* causing whirling disease in salmonids (mainly in the most susceptible fish host, rainbow trout), the infection severity was moderate, as infection trials were terminated 1 month p.e., prior to the appearance of severe clinical signs. Fish were anaesthetized with 200 mg/ml MS222 prior to blood sampling. The experiments were terminated with the application of a cervical cut on anesthetized fish.

### DNA extraction and qPCR assays

For DNA extraction from blood samples, DNeasy Blood & Tissue Kit (Qiagen, Germany) was used according to the manufacturer’s instructions. The amount and integrity of DNA was checked with 1% agarose gel electrophoresis and NanoDrop 2000c spectrophotometer (Thermo Scientific, USA).

Quantitative real-time PCR (qPCR) was used to detect the infection prevalence and to estimate the quantity of parasite in host blood. The amount of parasite (i.e. target) gene was normalized to the amount of fish (i.e. reference) gene. Target genes were the SSU rDNA of parasites *M*. *cerebralis* [27] and *M*. *pseudodispar* (developed and optimized in the present study), the reference genes were the insulin growth factor I (IGF-I) in salmonids [27] and glucokinase in cyprinid host species [28]. The specificity of qPCR assays was confirmed with DNA sequencing (S1 Text). Amplification conditions were: 10 min at 94°C, 40 cycles of 15 sec at 94°C and 60 sec at 60°C. To obtain optimal cycling conditions, single-run qPCR was applied for *M*. *cerebralis* samples, whereas multiplex qPCR was performed on *M*. *pseudodispar* samples. During optimization trials, we followed the multiplex qPCR set-up described by Kelley et al. for *M*. *cerebralis* [27], but it resulted in unsuccessful runs, therefore single-run qPCR was used for *M*. *cerebralis* with modified concentrations in reaction mixtures. The total volume of the reaction was 25 μl. The *M*. *pseudodispar*-specific multiplex qPCR contained 1x AmpliTaq Gold buffer (Life Technologies, Thermo Fisher Scientific, USA), 2.5 mM MgCl_2_, 300 μM dNTP mix (Fermentas, Thermo Fisher Scientific, USA), 400 nM of each primer (IDT, Belgium), 200 nM of the TaqMan probe (IDT, Belgium), 1.25 U AmpliTaq Gold DNA polymerase (Life Technologies, Thermo Fisher Scientific, USA) and 40–200 ng of template DNA. In the single-run qPCR of *M*. *cerebralis*, the components of the reaction were the same as for the multiplex qPCR, except the amount of probe (400 nM for SSU), the amount of DNA polymerase (0.75 U for SSU and IGF-I, respectively), and the amount of primers (200 nM for IGF-I). Reactions were run and analysed on a Rotor-Gene 6000 real-time rotary analyser (Corbett Life Science, Australia). Standard curves were obtained using 10-fold dilution series of gBlocks Gene Fragments, double-stranded synthesised DNA of the genes of interest (synthesised by IDT, Belgium), in triplicate reactions. The target genes, primers and fluorescent-labelled probes used for qPCR assays are listed in Table 1. The baselines and thresholds were manually set following visual examination of every run. Raw Ct (threshold cycle) values were calculated with the software Rotor-Gene Q version 2.3.1 (Qiagen, Germany). Ct values were not recorded when the curves did not reach the threshold value after 40 cycles. The descriptive values (slope, *y* intercept, coefficient of determination–R^2, etc.) of all qPCR runs are listed in S1 Table. The lowest gene copy number was 100 that was reliably detected in every run. Raw Ct data were normalised to the reference gene, and inter-run calibration was performed with the software qBasePlus [29] to eliminate the effect of amplification efficiency differences among different qPCR runs [30–31]. Calibrated normalised relative quantities (CNRQ values) were calculated, which represented the relative amount of the target gene (i.e. SSU rDNA) compared to the amount of reference genes used for normalisation (i.e. IGF-I and glucokinase, respectively).

**Table 1:** Primers and probes used for *Myxobolus cerebralis* and *M. pseudodispar-specific* qPCR assays.

Target gene	Primer name	Primer sequence (5’ → 3’)	Probe name	Probe sequence (5’ → 3’)	Amplicon size (bp)	GenBank reference	Reference
***M*. *cerebralis***							
IGF-I	tgIGF1-231f	CAGTTCACGGCGGTCACAT	tgIGF1-252p	/5HEX/CCGTGGTAT/ZEN/ TGTGGACGAGTGCTGC/3IABkFQ/	67	M95183	Kelley et al., 2004
	tgIGF1-297r	CCGTAGCTCGCAACTCTGG					
Myx18S	Myx18-909f	CTTTGACTGAATGTTATTCAGTTACAGCA	Myx18-953p	/56-FAM/ACCGGCCAA/ZEN/ GGACTAACGAATGCG/3IABkFQ/	178*	AF115253	Kelley et al., 2004
	Myx18-996r	GCGGTCTGGGCAAATGC					
***M*. *pseudodispar***							
Gluc	CgGluc-162f	ACTGCGAGTGGAGACACATGAT	cgGluc-185p	/Texas Red-X/AAGCCAGTGTCAAAATGCTGCCCACT/BHQ2/	69	AF053332	Gilad et al., 2004
	CgGluc-230r	TCAGGTGTGGAGCGGACAT					
Myx18S	Mp-736f	AAACAGAGTGCTCAAAGCAG	Mp-781p	/56-FAM/CCATGCTAC/ZEN/ AACATTCAAGCGTTCGC/3IABKFQ/ (ANTISENSE)	77	KU340983	present study
	Mp-812r	CACGATATGTGCGAATACACG					

Target gene was SSU rDNA of *M*. *cerebralis* and *M*. *pseudodispar* (Myx18S), reference genes were insulin growth factor-I (IGF-I) of salmonids and glucokinase (Gluc) of cyprinids. Probes labelled with HEX, FAM and TexasRed-X 5’ fluorochromes, respectively, ZEN internal quencher and a 3’ dark quencher (Iowa Black FQ; 3IABkFQ).

*original description by Kelley et al. (2004) refers to a 88 bp-long DNA fragment, however the valid size of the PCR product is 178 bp (S1 Text).

### Statistical analysis

Prior to statistical analyses, CNRQ values were log_10_ transformed to obtain normal distribution. To determine differences in prevalence, Chi-squared test was used. In the *M*. *cerebralis* experiment, Welch’s *t*-test was applied to compare parasite infection intensity (log_10_ CNRQ values) in brown trout and rainbow trout. In the *M*. *pseudodispar* experiments, ANOVA and Tukey’s *post-hoc* test was applied to compare differences of infection intensity among host species, except the samples at time point 1 month p.e., which were analysed by Welch’s *t*-test due to the absence of common bream samples. Statistical analyses were performed using the R program, version 3.3.1 [32] (available in S1 Dataset). The level of significance was determined at *p* < 0.05, and 'borderline' significance was identified in a case when *p* value was between 0.05 and 0.1.

## Results

### *Myxobolus cerebralis* exposure

**Fig 1:**
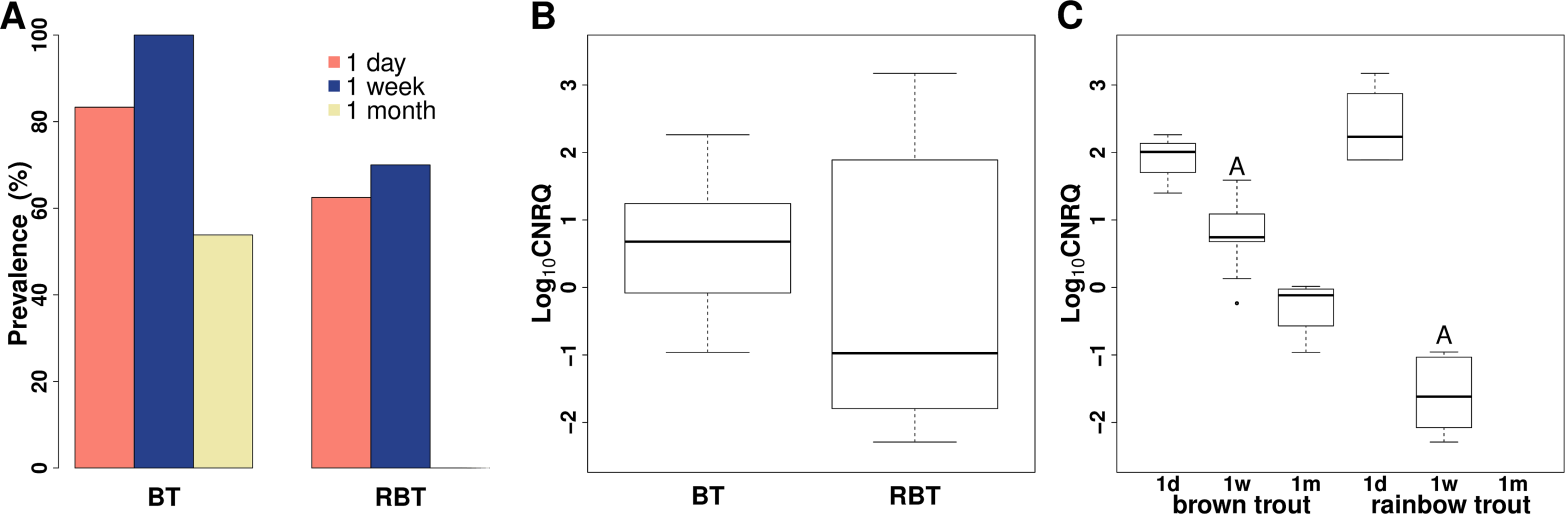
Prevalence and intensity of *Myxobolus cerebralis* in host blood. (A) Prevalence (in percentage) of the parasite in host species in the relation of sampling time. (B) Boxplot of parasite infection intensity in the examined host species. (C) Boxplot of infection intensity by host species and at different sampling points. Significant difference: A: *p* < 0.001. RBT: rainbow trout *Onchorhynchus mykiss*; BT: brown trout *Salmo trutta* m *fario*. Box-and-whisker plots: box: interquartile range; bold line in the box: median; whiskers: minimum and maximum values; point (circle): outlier. Log_10_CNRQ: log10 transformed, calibrated normalized relative quantities of parasite DNA based on qPCR measurements.

The *M*. *cerebralis*-specific qPCR described by Kelley et al. [27] refers to an 88 bp-long DNA fragment, however the valid size of the PCR product was 178 bp (confirmed with DNA sequencing; S1 Text). In the case of some individuals, the sampling of blood was not possible, mainly due to the small size of the fish (especially the salmonids, which were ~4 cm in length at the time of the first two sampling). Therefore the number of sampled fish was less than the number of fish exposed in some of the experimental groups (Table 2). The presence of *M*. *cerebralis* in blood samples was confirmed with qPCR in both trout species. qPCR assays were all negative for uninfected fish. The prevalence was significantly different between the two species (Chi-square test: *p* = 0.015); more brown trout specimens were *M*. *cerebralis* positive than rainbow trout (Table 2; S2 Table). One month p.e., the prevalence decreased below the detection limit of qPCR in rainbow trout (Fig 1A). Brown trout had higher overall infection intensity (i.e. higher CNRQ values), which showed a trend towards significance (Fig 1B). Infection intensity decreased over time and this trend showed a faster rate in rainbow trout (Fig 1C). One week p.e., significant difference was detected in the infection intensity between the two host species (Welch’s *t*-test: *p* < 0.001; Fig 1C). A significant difference was also detected in the samples collected 1 month p.e., a low intensity was observed in brown trout, however, no parasite DNA was detected in rainbow trout (Fig 1C).

**Table 2:** Descriptive statistics of *Myxobolus cerebralis* infection in the blood of salmonids.

Fish species		Time point (p.e.)	log_10_CNRQ			No. of infected/ sampled fish*	Prevalence (%)
			Min–Max	Median	SD		
Brown trout		1 day	1.4–2.26	2.01	0.440	5/6	83.33
		1 week	-0.23–1.59	0.74	0.580	9/9	100
		1 month	-0.96–0.02	-0.12	0.400	7/13	53.85
Rainbow trout		1 day	1.889–3.173	2.232	0.619	5/8	62.5
		1 week	-2.292 –-0.957	-1.619	0.580	7/10	70
		1 month	0	0	0	0/12	0

Data regarding infection intensity are based on log-transformed calibrated normalised relative quantities values (Log_10_CNRQ) obtained with qPCR; per fish species per sampling time point (post exposure, p.e.). Minimum and maximum intensity (Min–Max); standard deviance (SD).

0: no parasite detected.

* No. of sampled fish was equal or less than the No. of fish exposed, because in some cases, the sampling of blood was not possible, mainly due to the small size of fish.

### *Myxobolus pseudodispar* exposure trial #1

**Fig 2:**
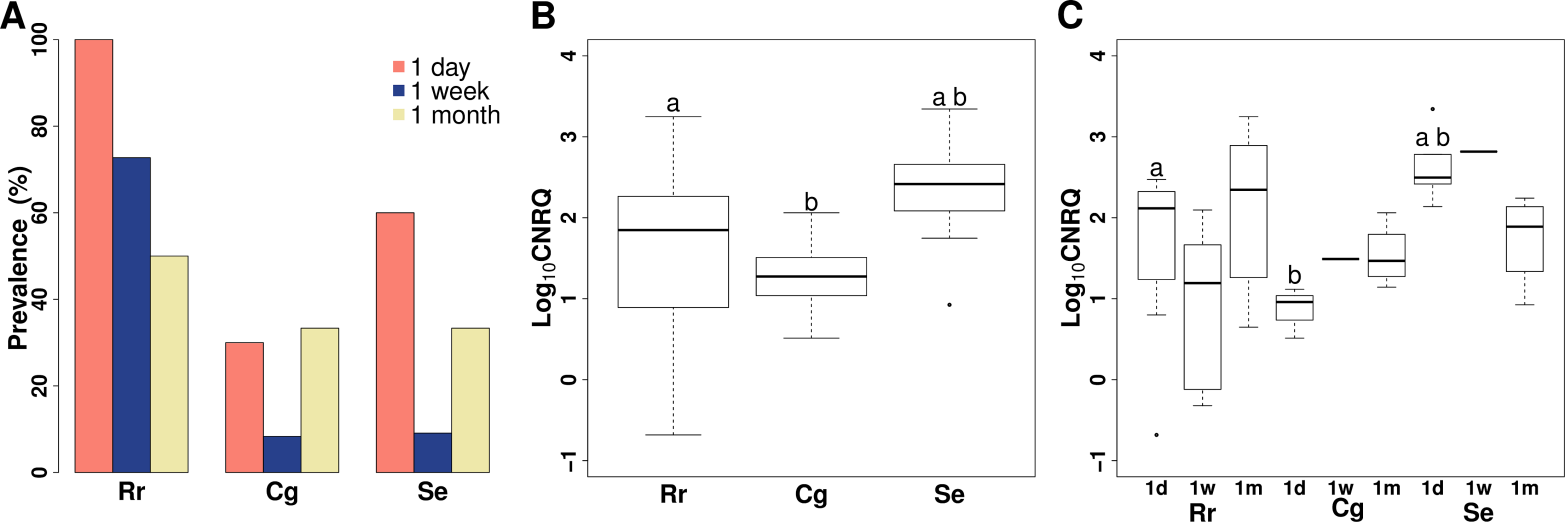
Prevalence and intensity of *Myxobolus pseudodispar* in host blood in exposure trial #1. (A) Prevalence of the parasite in host species in the relation of sampling time. (B) Boxplot of infection intensity in examined fish species. Significant differences: a: *p* = 0.043, b: *p* = 0.041. (C) Boxplot of infection intensity by fish species and at different sampling time. Significant or borderline differences: a: *p* = 0.087, b: *p* = 0.016. Rr: common roach *Rutilus rutilus*; Cg: gibel carp *Carassius gibelio*; Se: rudd *Scardinius erythrophthalmus*; Log_10_CNRQ: log10 transformed, calibrated normalized relative quantities of parasite DNA based on qPCR measurements.

The prevalence of *M*. *pseudodispar* infection was significantly different among host species (Chi-square test: *p* < 0.001). In common roach, higher infection prevalence was detected than in gibel carp and in rudd (Table 3; Fig 2A; S3 Table). qPCR assays were all negative for uninfected fish. Both the overall infection intensity and the intensity 1 week p.e, was the highest in rudd (Fig 2B). The infection intensity difference was significant between rudd and gibel carp (ANOVA & Tukey’s *post-hoc* test, overall: *p* = 0.041; 1 day: *p* = 0.016), and between rudd and common roach (overall: *p* = 0.043; “borderline significance” at 1 day: *p* = 0.087). The difference was not significant between common roach and gibel carp (Fig 2B and 2C). One week and 1 month p.e., the intensity differences were non-significant among fish species examined (Fig 2C). Time trend in *M*. *pseudodispar* infection was not observable; infection intensities have remained more or less constant until the end of experiment (Fig 2C).

**Table 3:** Descriptive statistics of *Myxobolus pseudodispar* infection in the blood of cyprinids.

Fish species	Time point (p.e.)	log_10_CNRQ			No. of infected/ sampled fish*	Prevalence (%)
		Min–Max	Median	SD		
**Exposure trial #1**						
Common roach	1 day	-0.68–2.47	2.12	0.99	10/10	100
	1 week	-0.32–2.10	1.19	0.97	8/11	72.73
	1 month	0.65–3.25	2.35	1.10	5/10	50
Gibel carp	1 day	0.51–1.12	0.96	0.31	3/10	30
	1 week	1.49–1.49	1.49	NA	1/12	8.33
	1 month	1.14–2.06	1.47	0.39	4/12	33.33
Rudd	1 day	2.14–3.34	2.50	0.41	6/10	60
	1 week	2.82–2.82	2.82	NA	1/11	9.09
	1 month	0.92–2.24	1.89	0.58	4/12	33.33
**Exposure trial #2**						
Common roach	1 day	0.78–1.83	1.33	0.43	6/9	66.67
	1 week	0.91–1.83	1.07	0.40	5/9	55.56
	1 month	0.11–2.79	1.54	0.87	8/10	80
Common bream	1 day	1.23–1.33	1.28	0.07	2/9	22.22
	1 week	0.91–1.69	1.30	0.55	2/9	22.22
	1 month	n.d.	n.d.	n.d.	n.d.	n.d.
Rudd	1 day	2.20–3.95	2.58	0.64	6/9	66.67
	1 week	1.79–5.62	2.74	1.36	6/9	66.67
	1 month	2.05–3.11	2.41	0.39	4/10	40

Data regarding infection intensity are based on log-transformed calibrated normalised relative quantities values (Log_10_CNRQ) obtained with qPCR; per fish species per sampling time point (post exposure, p.e.). Minimum and maximum intensity (Min–Max); standard deviance (SD).

n.d.: no data available. NA: not applied

* No. of sampled fish was equal or less than the No. of fish exposed, because in so––me cases, the sampling of blood was not possible, mainly due to the small size of fish.

### *M*. *pseudodispar* exposure trial #2

**Fig 3:**
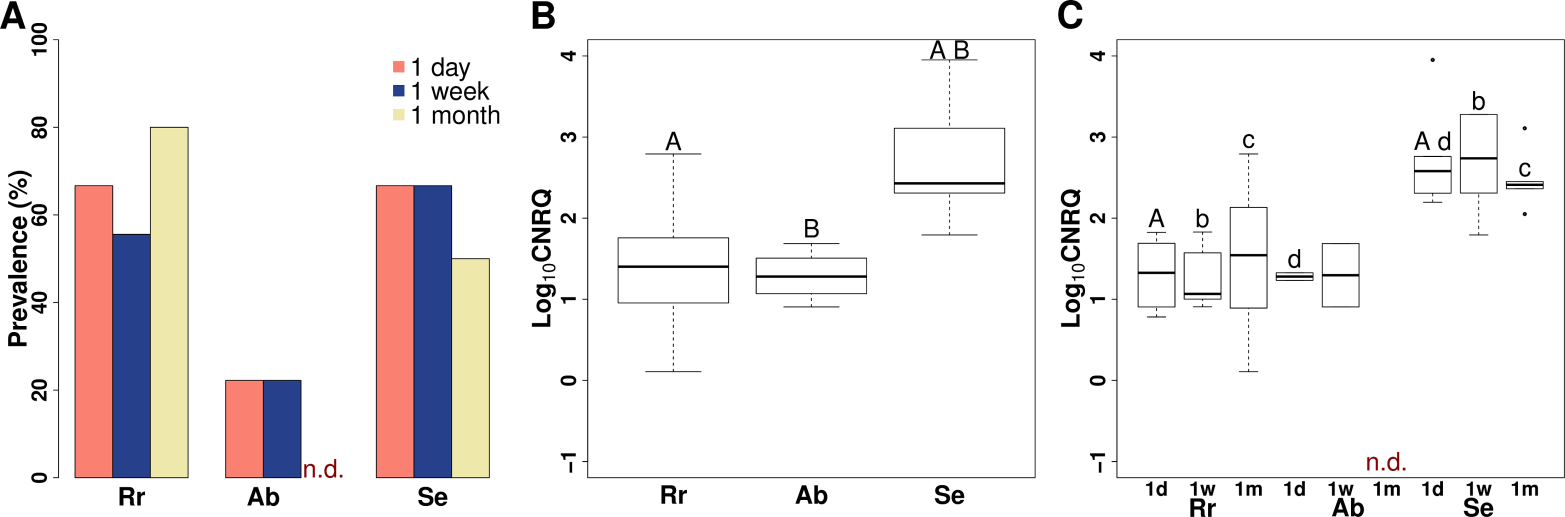
Prevalence and intensity of *Myxobolus pseudodispar* in fish blood in exposure trial #2. (A) Prevalence of the parasite in host species in the relation of sampling time. (B) Boxplot of infection intensity in different fish species. Significant differences A: *p* < 0.001, B: *p* = 0.003. (C) Boxplot of infection intensity by fish species and sampling time. Letters above boxes indicate significant differences: A: *p* = 0.002, b: *p* = 0.035, c: *p* = 0.020, d: *p* = 0.015. Rr: common roach *Rutilus rutilus*; Ab: common bream *Abramis brama*; Se: rudd *Scardinius erythrophthalmus*; n.d. = no data; Log_10_CNRQ: log10 transformed, calibrated normalized relative quantities of parasite DNA based on qPCR measurements.

The prevalence among hosts was significantly different (Chi-square test: *p* = 0.007). Lower prevalence was detected in common bream than in the other two cyprinid species (Table 3; Fig 3A; S3 Table). The overall infection intensity was significantly higher in rudd than in common bream or common roach (Fig 3B): rudd vs. common roach (overall: *p* < 0.001; 1 day: *p* = 0.002; 1 week: *p* = 0.035; 1 month: *p* = 0.020), rudd vs. common bream (*p* = 0.003; 1 day: *p* = 0.015). Common roach and common bream possessed similar level of infection intensity with no significant difference (Fig 3B). Infection intensity at every time point was very similar in common roach and common bream. Significant difference was detected between rudd and the other two hosts only (Fig 3C). Time trend in infection intensity was not observable, similarly to *M*. *p*. experiment #1 (Fig 3C). The visual confirmation of the presence of early parasite stages in blood smears was unsuccessful.

## Discussion

In the present study, we intended to take one step forward in the understanding of the nature of host specificity. Based on the studies on a handful of myxozoan species, we assume that most histozoic myxozoans migrate through several organs and tissues until they reach the place of sporogony [6]. Using blood and the host’s recirculation system seems to be a logical option, as that would be the easiest way to get passively transported from the portal of entry to the tissue where spore formation takes place [6]. The most known example for this phenomenon was observed for *Sphaerospora* sensu stricto (s. str.) species. *Sphaerospora dykovae* Lom and Dyková, 1982 responsible for swim bladder inflammation of common carp, and other related *Sphaerospora* species possess “blood stages”, that cause inflammation in the swim bladder of carp fry, although the place of sporogony is elsewhere (e.g. in the kidneys for *S*. *dykovae*, or gills for *Sphaerospora molnari* Lom, Dyková, Pavlásková et al., 1983) [6,33,34]. For the spherosporids, these developmental stages also proliferate in blood, they are extrasporogonic stages, which are in a “schizogonic phase” in the blood, where they greatly increase in number. These stages are easily observable in blood smears, thereby the cell-organisation of several different type of blood stages belonging to various *Sphaerospora* species have been well documented [33,35,36].

The study of the entire intrapiscine development of *M*. *cerebralis* based on histology and electron microscopy by El-Matbouli et al. [13] included the investigation of blood. Although the blood smears of all experimentally exposed fish were examined, no evidence of the parasite occurring in blood was detected and thereby, no potential contact of the parasite with immunocompetent cells, which could trigger an immune response. In our study, the blood smears of fish from *M*. *pseudodispar* exposure trial #2 were examined. As preliminary exposure results obtained using parasite-specific nested PCR assays indicated, the parasite quantity of *M*. *pseudodispar* in blood was often 100-fold higher than for *M*. *cerebralis* [37], therefore we presumed that the opportunity was much higher to observe the blood stages of *M*. *pseudodispar* especially in heavily infected specimens. Counter to this finding, no cell-in-cell stages resembling the known myxozoan blood stage were detected, even in heavily infected specimens. However, cell-formation may account for this discrepancy, *Myxobolus* blood stages could have a different shape, size, or cell-organization than the known sphaerosporids. If this is the case, parasite-specific *in situ* hybridization should have been performed instead of a modified Giemsa staining. On the other hand, it is likely that the quantity of *Myxobolus* blood stages was below the detection limit of microscopy. This is not necessarily an exceptional case, certain pathogenic bacteria stains (e.g. *Mycobacterium* sp.) could not be detected microscopically using traditional techniques either. Although with PCR and isolation methods, their presence in host could be confirmed [38].

Other myxozoans than sphaerosporids, we assume that no myxozoans were seen or detected with traditional microscopy in blood. These developmental stages, if they are present in blood, do not, presumably, replicate and develop in the vascular system, they just simply use the blood stream for transport. The presence of a non-sphaerosporid species in blood was observed by Holzer et al. [39] using molecular techniques. Besides characterizing new members of *Sphaerospora* s. str. in common carp, gibel carp and goldfish, the authors reported that about 11% of the blood stages detected with PCR were *Myxobilatus gasterostei* (Parisi, 1912), a renal, intratubular parasite of the three-spined stickleback *Gasterosteus aculeatus*. Holzer et al. [39] did not observe *Myxobilatus*-type spores in any of the cyprinids studied, nor did they find any non-sphaerosporid-like blood stages in the examined blood smears. They concluded that the development of the stickleback-specific myxozoan in the unsuitable hosts (i.e. carp and goldfish) was probably arrested before spore formation could take place.

The early histology and electron microscopy-based study of *M*. *cerebralis* did not find evidence for the presence of the parasite in blood during the intrapiscine development [13]. Unlike expectations, our results, based on qPCR, proved that *M*. *cerebralis* in the early stage of development is present in the blood of both rainbow trout and brown trout. El-Matbouli et al. [13] asserted that if *M*. *cerebralis* is not present in blood, then the migration of the parasite via the nervous system to the cartilage was reasonable, and by choosing this route, the parasite could completely avoid blood contact, and thereby, immunocompetent cells. Kallert et al. [25] found, as a result of the *in vitro* inhibition assays, that *M*. *cerebralis* seems to lack any adaptation to blood immune factors, even its sporozoites were attacked by sera from either susceptible or more resistant rainbow trout strains. They concluded if *M*. *cerebralis* migrates along peripheral nerves to the central nervous system of fish, it would never get contact with the blood immune system and if it does, it might be destroyed. So in this species, there might have been a trade-off not to evade the bloodstream, but to invest in higher numbers of sporoplasm secondary cells (e.g., 64 in *M*. *cerebralis* instead of 8 in *M*. *pseudodispar*) by simply changing migration behaviour. If the route via the bloodstream is a “dead end” for the development of *M*. *cerebralis*, it might explain why the infection intensity decreased rapidly in both brown and rainbow trout over time during our experiment, and why no parasite DNA was detected in rainbow trout 1 month p.e. As peripheral nerves were not examined in our study, we cannot confirm the “real route” of intrapiscine migration, but we found no evidence allowing for the exclusion of this possibility either. Another explanation for the decrease of the infection intensity is the timing and nature of the development. El-Matbouli et al. [13] concluded that 4 to 24 days p.e. *M*. *cerebralis* stages were migrating to the place of sporogony (i.e., cartilage of central nervous system, CNS), and from day 20, parasite stages could be observed in head cartilage. Ifso, it is also likely that the number of migrating stages decreased in blood, as they were leaving the blood stream and migrated to the CNS. It is known, however, that blood stages of myxozoans may leave the blood to migrate to sporogonic site, but later they may return to cause systemic infection. For *Ceratonova shasta*, it has been proven that parasite levels in blood increased 4 days p.e. and remained at a consistent level until the second week, but then the parasite abundance increased further and coincided with host mortality caused by the parasite accumulation in intestine [40]. This could be the case for *M*. *pseudodispar*, as this parasite likely spreads through skeletal muscle over time and may re-enter blood system. But it also explains the opposite case of *M*. *cerebralis*, for which the ossification of head cartilage may prevent the re-infection of blood in a later stage of development. In rainbow trout, the decrease of infection intensity was more rapid than in brown trout. We propose that there might be a correlation between the severity of symptoms and the infection intensity in blood. In rainbow trout, heavy clinical signs are expected (especially in the Kamloops strain used for the experiment), with mild to no symptoms occurring in brown trout. Previous studies indicated that the severity of infection correlates with the intensity of infection measured, based on the number of parasite myxospores [7,41]. The level of infection intensity in blood 1 day p.e. was almost equal in both trout species. The rate of decline in infection intensity in blood differed considerably in the two fish species and could be related to host susceptibility differences. On the other hand, brown trout had a more remarkable cellular immune response by eosinophilic granular leucocytes than rainbow trout [10], which may explain these differences between the highly and less susceptible host species. Several other factors could influence the infection intensity, including the level of fish inbreeding, as confirmed by previous studies [42,12].

The intrapiscine development and host susceptibility of *M*. *pseudodispar* is less-studied than those of *M*. *cerebralis*. Exceptionally high levels of genetic variability among the SSU rDNA of *M*. *pseudodispar* isolates have been reported in several cases [5,15,17,43]. Experimental findings demonstrated the sensitivity differences among putative hosts, and correlation was detected between host specificity and the phylogenetic relationship of parasite isolates [15]. Forró & Eszterbauer [15] explained the unique phylogenetic position and host range of certain parasite isolates with host-shift and distinguished primary (i.e. original host, common roach), secondary (i.e. a host with lower infection intensity and prevalence; e.g. common bream) and unsusceptible hosts (i.e. no spore formation occurs; e.g. gibel carp) [15]. The prevalence and infection intensity was characterized based on the measured number of parasite myxospores in the skeletal muscle of fish. In the present study, we examined the same host species to see whether susceptibility differences are detectable in the early stage of development, or if host specificity is determined only at a later stage e.g., in the course of sporogony. Regarding the findings of both experimental exposures with *M*. *pseudodispar* lineage GER, the highest prevalence in blood was not accompanied by the highest infection intensity. The highest prevalence was found in the primary host, the common roach, and the significantly highest infection intensity was detected in rudd. Forró & Eszterbauer [15] confirmed the lack of sporogony in rudd, which could explain these findings, and indicates that the parasite infection is aborted right before or during sporogony. Therefore rudd are supposed to be a “dead end” in the life cycle of *M*. *pseudodispar* lineage GER. It is possible that the parasites in rudd do not receive the adequate cue for directing them to the site of sporogony and hence, they accumulate in the blood. In susceptible hosts, parasites probably exit the bloodstream at the site of spore formation.

A similar phenomenon was observed in the definitive host of *M*. *pseudodispar* [43]. Oligochaete hosts, *Tubifex tubifex* lineage V and *Limnodrilus hoffmeisteri* were positive in a *M*. *pseudodispar*-specific PCR, but in most cases, the release of mature, fish-infecting actinospores could not be detected. The authors concluded that these oligochaete species/strains likely serve as “biological filters” as they remove myxospores from the sediment without producing actinospores [43].

Interestingly, in the non-susceptible host gibel carp, the prevalence and intensity of infection were very similar to those observed in the secondary host, common bream. Previous studies indicated that *M*. *pseudodispar* does not develop myxospores in gibel carp [15], therefore, we also expected that the parasite would be absent in the blood. This was not the case, our findings supply further evidence, that host specificity is not determined during the “migration stage”of development, thus early parasite stages in the bloodstream are not host specific, similar to the actinospores penetrating the host.

When compared, the infection dynamics of the two species examined, showed remarkable differences. While the infection intensity of *M*. *cerebralis* rapidly declined, *M*. *pseudodispar* infection remained more or less constant throughout the entire experiment. The observed differences in the time trend may be related to their dissimilar pathogenicity, the discrepancies in the adaptation to blood immune factors, and/or the site of spore formation. As *M*. *pseudodispar* is non-pathogenic, the selective pressure on resistance development by the host should be rather low. It is likely that hosts did not acquire adaptive immunity against the parasite. This is probably not the case for *M*. *cerebralis*, that could be another reason why the declining infection intensity was observed in the blood.

## Conclusion

Our findings, based on qPCR analysis, supplied essential details on the host specificity of the examined histozoic *Myxobolus* species. We proved that *M*. *cerebralis* does enters the bloodstream in the early stage of infection. The infection intensity in blood decreased over time for both examined salmonid hosts, but the most remarkable decline was detected in rainbow trout, in which the parasite cause the most severe symptoms. For the non-pathogenic *M*. *pseudodispar* lineage GER, instead of the original host common roach, the significantly highest infection intensity was detected in rudd a likely “dead end” for parasite development. We found impressive disparity in the infection dynamics of the two examined *Myxobolus* species. The observed divergence in time trend may be due to their distinct pathogenicity or site preference. Our findings supply further evidence that host specificity is not determined during the “migration stage”of development, thus early parasite stages in blood are not host specific, similar to the fish-invading actinospores.

## Supporting information

S1 Table Efficiency data of qPCR assays. (XLS)

S2 Table CNRQ values obtained with *Myxobolus cerebralis*-specific qPCR and subsequent data analysis with the software qBasePlus. CNRQ: calibrated normalized relative quantities of *M*. *cerebralis* DNA.
(XLS)

S3 Table CNRQ values obtained with *Myxobolus pseudodispar-*specific qPCR and subsequent data analysis with the software qBasePlus. CNRQ: calibrated normalized relative quantities of *M*. *pseudodispar* DNA.
(XLS)

S1 Text DNA sequences of PCR products obtained with parasite-specific PCR assays. (TXT)

S1 Dataset *R* codes of statistical analyses. (ZIP)
